# A Human-Relevant 3D In Vitro Platform for an Effective and Rapid Simulation of Workplace Exposure to Nanoparticles: Silica Nanoparticles as Case Study

**DOI:** 10.3390/nano10091761

**Published:** 2020-09-06

**Authors:** Luisana Di Cristo, Fabio Boccuni, Sergio Iavicoli, Stefania Sabella

**Affiliations:** 1 Istituto Italiano di Tecnologia, Nanoregulatory Platform, Drug Discovery and Development Department, 16163 Genova, Italy; stefania.sabella@iit.it; 2 Italian Workers’ Compensation Authority (INAIL), Department of Occupational and Environmental Medicine, Epidemiology and Hygiene, Monte Porzio Catone, 00078 Rome, Italy; f.boccuni@inail.it (F.B.); s.iavicoli@inail.it (S.I.)

**Keywords:** nanomaterial risk assessment, occupational exposure assessment, inhalation toxicity, aerosol system, silica nanoparticles

## Abstract

In this contribution, we show the suitability of a 3D airway model, when coupled with a nebulizer system, for simulating workplace exposure to nanoparticles. As a proof of concept, workplace exposure to silica nanoparticles was experimentally measured in an occupational facility where nanoparticles are produced weekly, and compared with the official limit value for bulk silica materials. These values of potential exposure were simulated in a 3D airway model by nebulizing low doses (from 0.90 to 55 µg/cm^2^) of silica nanoparticles over a prolonged period (12 weeks of repeated exposure, 5 days per week). Overall, the results suggest the efficiency of the defense mechanisms of the respiratory system and the clearance of the breathed silica nanoparticles by the mucociliary apparatus in accordance with the recent in vivo data. This in vitro platform shows that the doses tested may correlate with the occupational exposure limit values. Such relationship could provide regulatory-oriented data useful for risk classification of nanomaterials.

## 1. Introduction

Nowadays there is still a lot of interest in the potential adverse health effects associated with the exposure to engineered nanomaterials (ENM) from the scientific, public and government communities. This interest is based on little knowledge on quantities of ENMs to which workers could be potentially exposed during their entire occupational lifetime due to ENM release into the environment [1,2]. It has been demonstrated that workplace exposure primarily occurs due to the presence of aerosol containing ENMs in their pristine or aggregate forms; therefore, the predominant route to consider is inhalation. Based on recent epidemiological studies on ultrafine particles, chronic exposure in occupational settings has been related to pulmonary inflammation and its related consequences [1]. However, to date, there is no evidence that shows that such effects have ever occurred in workers during the handling of specific ENMs [1]. However, from the side of quantification of exposure limits of ENMs, available data are very scarce, and currently, no official occupational exposure limits (OELs) have been established for many types of ENMs [3,4].

Here, we propose an in vitro platform (3D airway model coupled with a nebulizer system) where proper experimental conditions attempt to simulate the route of exposure (i.e., lung) and doses derived from real-world exposure scenarios (i.e., dose values that refer to repeated exposure simulating a worker lifetime of 40 years). By testing this in vitro platform on ENMs, the final aim is to provide regulatory-oriented data to support the risk assessment of nanomaterials. In this respect, to simulate inhalation, we focused on the lung. Lungs consist of a series of structural and functional barriers (nasopharyngeal, tracheobronchial, and pulmonary regions) that deal with inhaled ENMs before being conducted deeply into the lung [5,6]. As a case study for the platform, we selected amorphous silica nanoparticles (SiO_2_ NPs). Due to the development of silicosis by crystalline silica, silica in this form is one of the most common and well-studied occupational hazards [7]. In general, amorphous silica is considered to be less harmful than its crystalline counterpart [7,8]; however, recent toxicity data have demonstrated that the adverse effects are linked more to surface chemistry, specifically to silanol disorganization, than to crystallinity state [9]. A recent review focusing on the health surveillance of workers involved in the production of metal oxide ENMs highlighted that workers exposed to the manufacturing of SiO_2_ NPs may develop some side effects. However, such effects cannot be strictly related to SiO_2_ NPs due to heterogeneous factors of the studied environment (e.g., more than one type of metal ENMs produced in the manufacturing facility and the presence of solvents in the synthetic procedures) [10]. Another recent study (divided into parts I and II) performed in parallel a workplace exposure characterization of SiO_2_ NPs and biomonitoring assessment of workers potentially exposed. This coupled study showed pieces of evidence of the presence of SiO_2_ NP emission in the workplace and, as a potential consequence, an early still-reparable genotoxicity in workers exposed during the manufacturing processes. Nevertheless, also in this case, the authors concluded that the biomarkers monitored cannot discriminate between the nanospecific effect and other factors (such as solvents or chemicals employed in the facility) [11,12].

On the side of in vivo animal contributions, in a 5-day inhalation study, Art et al. demonstrated that lung clearance of SiO_2_ NPs plays an important role in determining toxicity in rats. Indeed, silicon clearance from the lungs of exposed animals was rapid and complete and the inflammation transient during the 3-month observation period [13]. Sayes et al., who performed a short-term inhalation study (3 days) on rats, reported that even high concentrations of particles did not provoke any inflammatory or genotoxic responses in the lungs [14]. Additionally, other authors demonstrated that although there were minimal toxic changes, subacute inhalation (28 days) to silica had no toxic effects on rats’s lung at the used concentrations and selected time points [15]. Lastly, in a long-term inhalation experiment, Sutunkova et al. demonstrated a very low systemic toxicity and negligible pulmonary effects [16]. Overall, these in vivo data reported no significant lung toxicity after silica exposure primarily due to NP clearance. This indication could support the hypothesis of epidemiological studies which indicate that the weak toxicity evidenced on the worker population could be multifactorial. Indeed, this scarce toxic effects are due to many components involved in the production processes and, not necessarily due to nanoparticles. However, the paucity of both epidemiological and in vivo studies on ENMs suggests caution and requires a more detailed investigation in both directions.

In this complex scenario, the development of three-dimensional (3D) in vitro models coupled with a nebulizer system (as explained above) that can mimic tissue structure and in vivo functionalities could provide great benefits to answer, although partially, the above questions raised by in vivo and epidemiological studies, especially regarding toxicity testing [17,18]. Advanced in vitro 3D models resembling the lung are currently available, as two-dimensional (2D) models lack the complexity of physiological systems and long-term exposure studies [18,19]. Pulmonary interface is mimicked by using a 3D (airway/lung) model culture at the air–liquid interface (ALI), which more closely resembles the in vivo lung epithelium, where the apical surface is exposed to air and the basal surface of the cells is in contact with the liquid culture medium. Therefore, 3D in vitro models coupled with ALI exposure could be the alternative to overcome gaps between the simplistic in vitro cultures and the in vivo models. Over the last few years, only a few works have combined the use of 3D airway/lung models and a nebulizer system for long-term experiments [20,21,22]. In particular, the exposure time spans from 3 to 5 weeks. In the present contribution, we push forward this setup, prolonging the exposure time up to 12 weeks of repeated exposure to SiO_2_ NPs. Preliminary data on the tissue barrier integrity of the 3D in vitro model following SiO_2_ NP exposure were shown at the end of the selected time points in weeks (w): w2, w4, w6, w8, and w12.

## 2. Materials and Methods

### 2.1. Chemicals and Reagents

All chemicals and reagents used were obtained from Sigma-Aldrich (Milan, Italy), unless otherwise stated.

### 2.2. Silica Nanoparticles

Commercially available amorphous silica nanoparticles (SiO_2_ NPs, 50 nm) were obtained from HiQ-Nano S.r.l. (Arnesano, Italy). SiO_2_ NPs were dispersed in endotoxin-free water at a concentration of 1 mg/mL and then tested for the presence of endotoxin by limulus amebocyte lysate (LAL) assay (Pierce, Thermo Scientific, Milan, Italy). Acceptable and insignificant levels of endotoxin (0.005 ± 0.004 EU) were found according to US Food and Drug Administration (FDA) guidelines [23].

### 2.3. Aerosol Characterization

The Vitrocell^®^ Cloud ALI Starter Kit (Vitrocell^®^, Waldkirch, Germany) was used to produce SiO_2_ NP aerosol, and its deposition was quantified by the incorporated quartz crystal microbalance (QCM) and calculated as mass per surface area (μg/cm^2^) [22]. This system is specifically designed for dose-controlled and spatially uniform deposition of liquid aerosols on cells cultured at ALI conditions as it is equipped with an Aeroneb^®^ Pro nebulizer (span of 4.0 to 6.0 µm), incorporating the OnQ aerosol generator, which produces precisely controlled droplets [24]. The exposure chamber of the Vitrocell^®^ system is composed of two wells. One well is dedicated to the exposure of cells to aerosol, and one to the assessment of the real-time mass deposition in the entire module by means of a quartz crystal microbalance (QCM, operated at 5 MHz, detection limit: 0.09 μg/cm^2^) that is placed in such well. The QCM is connected to the Vitrocell^®^ microbalance software, where a microbalance controller is used to follow the QCM measurements during the treatment. In particular, the acquisition of the mass was taken at the end of the cell treatment every day. Simultaneously, we performed scanning electron microscopy (SEM) of the empty well insert in the module after the nebulization to have a qualitative inspection of the deposited materials by means of a JEOL JSM6490LA microscope (Tokyo, Japan).

### 2.4. Exposure Conditions

To characterize the potential adverse effect of SiO_2_ NPs in an environment that simulates the occupational setting, the 3D human airway model (see below) was exposed to aerosolized SiO_2_ NPs every working day from Monday to Friday (5 times per week) up to 12 weeks. An amount of 1 mg/mL of SiO2 NPs and a nebulizer flux of 1 min for nebulizing 125 µL of suspension were settled to obtain different cumulative doses, ranking from a minimum of 0.90 ± 0.10 µg/cm^2^ (1 nebulization) to a maximum of 55.25 ± 0.22 μg/cm^2^ (after 12 weeks of repeated daily exposure). Deposition of endotoxin-free water was used as negative control.

### 2.5. 3D Airway Model

A 3D mucociliary tissue model of primary human bronchial epithelium (MucilAir™) was purchased from Epithelix (Epithelix Sàrl, Geneva, Switzerland). Cells were cultured at ALI in 24-well Transwell inserts (6.5 mm diameter, 0.4 μm pore size, Corning Incorporated, Milan, Italy) following the supplier’s instructions. Upon receipt, the MucilAir™ models were maintained in culture for 3 days prior to performing the experiments. The basal culture medium was refreshed every 2–3 days.

### 2.6. Cell Viability

At the end of each selected week, the viability of the 3D airway model was assessed using a well-characterized assay, the AlamarBlue (resazurin) assay [22,24,25,26,27,28]. Resazurin is a non-fluorescent, membrane-permeable, blue compound that could be reduced by metabolically active cells into resorufin, a fluorescent pink dye that is extruded into the cell culture medium. Tissues were incubated for 1 h with a fresh medium supplemented with resazurin, added to both basolateral and apical compartments. Fluorescence measured at 572 nm, were performed on the medium of the apical chamber by means of a Tecan Spark multimode microplate reader (Tecan Italia S.r.l., Milan, Italy). Cell viability was calculated as a percentage (%) relative to the untreated (negative) control cell cultures. For positive control, cell cultures were exposed to 0.3% Triton X-100 in DPBS for 24 h.

### 2.7. Barrier Integrity Characterization

Before and after SiO_2_ NP aerosolization, the integrity of the tissue was measured by transepithelial electrical resistance (TEER) using an epithelial voltohmmeter (Millicell-ERS voltmeter, Millipore, Milan, Italy) as described in several publications [22,25,26,27,28]. For the analysis of the expression of constitutive membrane proteins, the Western blot method was used according to the protocol previously described [22]. Anti-E-cadherin (1:1000, Cell Signaling Technology, Milan, Italy), occludin-1 (1:1000, Abcam, Cambridge, UK), and anti-GAPDH (1:1000, Cell Signaling Technology) were used as antibodies. To analyze the barrier integrity at the microscope level, cells were processed both for transmission electron microscopy (TEM) and for laser scanning confocal microscopy (LSCM) as reported in our contribution, Di Cristo et al. [22]. For LSCM, anti-mucin 5AC antibody (Abcam 3649, 1:100 dilution) was used for staining goblet cells, anti-alpha tubulin (acetyl K40) antibody (Abcam 24610, 1:200 dilution) was used for staining cilium cells, and anti-zonula occludens (ZO-1) antibody (Abcam 216880, 1:200 dilution) was used for staining the tight junction. Hoechst 33342 (1:1000 dilution) was added for nucleus staining.

### 2.8. Measurements of Mass Concentration of SiO_2_ NPs in the Workplace

SiO_2_ NPs were produced in HiQ-Nano laboratory setting by synthesis in liquid phase and following washing and drying processes to obtain powders of 50 nm (average size) and two surface charges (positive and negative) [29,30]. Personal samplers (mod. Sioutas, SKC Inc., Eighty Four, PA, USA) equipped with a pump (9 L/min flow) were used to collect particles from 250 to 2500 nm (in five stages: >2500 (to 10,000), 1000–2500, 500–1000, 250–500, and <250 nm). Two different Sioutases were involved for parallel sampling for gravimetric analysis, one collecting nanomaterials in the worker’s personal breathing zone (PBZ) during production and the other one in the background. The total sampling time represented a typical working week of SiO_2_ NP production. Mass concentration of the collected particulate matter was calculated by weighting filters before and after the sampling (average of three weighting operations) by an analytical scale (mod. XS105, resolution = 0.01 µg). By subtracting the background value from the PBZ ones, with reference to the backup filter (<250 nm), the concentration of particulate matter with an aerodynamic diameter < 250 nm (PM_0.25_), to which the worker could be exposed in the operating conditions, was obtained [11,12].

## 3. Results and Discussion

**Figure 1:**
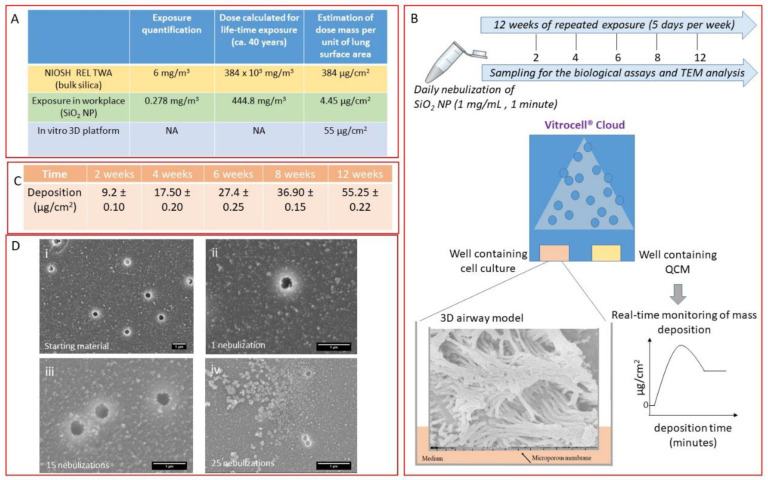
(**A**) Table reports values referring to the occupational exposure information for silica nanoparticles/bulk silica materials and the maximal dose deposited in our system. Yellow row: exposure limit available for the amorphous silica (bulk) reported by NIOSH (NIOSH Publication No. 2005-149) and its conversion to a dose mass per unit of lung surface area. Green row: upper limit of SiO_2_ nanoparticles (NPs) derived from the samplings in a manufacturing facility considering 40 years of worker exposure lifetime (experimentally measured). Blue row: maximal dose deposited during the 12 weeks of exposure in our 3D platform. (**B**) Schematic representation of the exposure condition and time interval of the treatment. (**C**) Table shows the average deposition (mean ± standard deviation) of aerosolized SiO_2_ NPs expressed in µg/cm^2^ measured by the quartz crystal microbalance (QCM) at different exposure time points (2, 4, 6, 8, 12 weeks). (**D**) Representative SEM images of SiO_2_ NPs before (**i**) and after nebulization at 1 (**ii**), 15 (**iii**), and (**iv**) 25-repeated exposure times, respectively. Scale bar: 1 µm.

As lack of realistic doses for both in vitro and in vivo studies is an important drawback for nanoregulatory testing [31], we applied doses, which refer to experimental levels of SiO_2_ NPs, derived from a production facility in which workers are involved in a daily synthesis of SiO_2_ NPs for one month (see below). This may be relevant in defining a correlation between the real environmental exposure of the workers and the response of the in vitro 3D platform. The amount of SiO_2_ NPs in the emission zones was quantified during the production process. In particular, workers who were involved weekly in the synthesis of SiO_2_ NPs with different average sizes (25 nm, 50 nm, and 100 nm) were considered to measure their potential inhalation exposure. Samplings of the particles emitted during the synthesis process of these nanoparticles were performed to collect the particulate matter with an aerodynamic diameter < 250 nm (PM_0.25_). The calculated value of the mass concentration in the worker’s personal breathing zone was 0.278 mg/m^3^ [12], representing the upper limit of the mass of SiO_2_ NPs to which the worker was potentially exposed during a week of activity (Figure 1A, green row). When compared with available official values, this upper limit appears to be below the OEL for amorphous silica (not in nanoform), which is 6 mg/m^3^ (Figure 1A, yellow row) [32]. Such latter value corresponds to the allowed human inhalation exposure to amorphous silica per day and has been approximately converted by Murugadoss et al. into a dose expressed in μg/cm^2^, which is found to be 384 μg/cm^2^ when considering a working exposure lifetime of 40 years. To harmonize the unit expression of the doses in μg/cm^2^ (considering 40 years of exposure), the conversion of the experimental upper limit value, using Murugadoss’s approach, results in a dose of 4.45 μg/cm^2^ (Figure 1A, green row). This harmonization also allows us to compare these values with the dose deposited by the 3D in vitro platform implemented in this work (see below).

The 3D platform contains an airway model that is a fully differentiated bronchial epithelial cell tissue, MucilAir™ (Epithelix Sàrl, Geneva, Switzerland), reconstituted from primary human cells (Figure 1B). This model can recapitulate the in vivo mucociliary response to a respiratory insult and is suitable for long-term studies; therefore, it is a very useful tool for simulating biological response to occupational exposure to nanomaterials. To simulate occupational aerosol exposure to SiO_2_ NPs, the Vitrocell^®^ Cloud ALI Starter Kit (Vitrocell^®^, Waldkirch, Germany) was used as it is a well-known nebulizer system able to deliver to the cells a very low amount of the tested compound [22]. A schematic representation of the 3D in vitro platform (the 3D airway model along with the nebulization process and the experimental conditions applied in the study) is shown in Figure 1B. To the best of our knowledge, this is the first time where a 3D airway model is exposed to SiO_2_ NPs for 12 weeks (5 days per week). Here, 125 μL of SiO_2_ NP suspension at a concentration of 1 mg/mL was nebulized (nebulizer flux of 1 min) every working day for 12 weeks (5 times per week). The deposited masses, as measured by the QCM present in one of the wells of the Vitrocell^®^ Cloud module, ranged from a single dose of 0.90 μg/cm^2^ to a final deposited dose of ca. 55 μg/cm^2^ upon 12 weeks of exposure (Figure 1C).

Moreover, SEM analysis was used to characterize SiO_2_ NP suspension before and after the aerosolization process (Figure 1D). The analysis showed that the aerosolization process did not induce any changes in the morphology of the deposited particles as no significant changes were observed compared with the starting material. Additionally, SEM images highlighted a dose-dependent deposition of the materials and the presence of small aggregates. Interestingly, the maximum dose deposited by the 3D in vitro system was settled in order to be consistent with the upper limit value (as experimentally detected) and with the official OELs (Figure 1A, blue row).

The airway barrier plays a crucial role as the first line of defense against inhaled particulate matter, thanks not only to the mucociliary system but also to the tight junction (TJ) formation and maintenance, which represent a dynamic structure governing the paracellular permeability of the epithelium [33]. TEER measurement, commonly used to assess the integrity and permeability of the barrier as an indirect measurement of the formation of the TJ [22,24,27,28], was monitored for all the 12 weeks.

**Figure 2:**
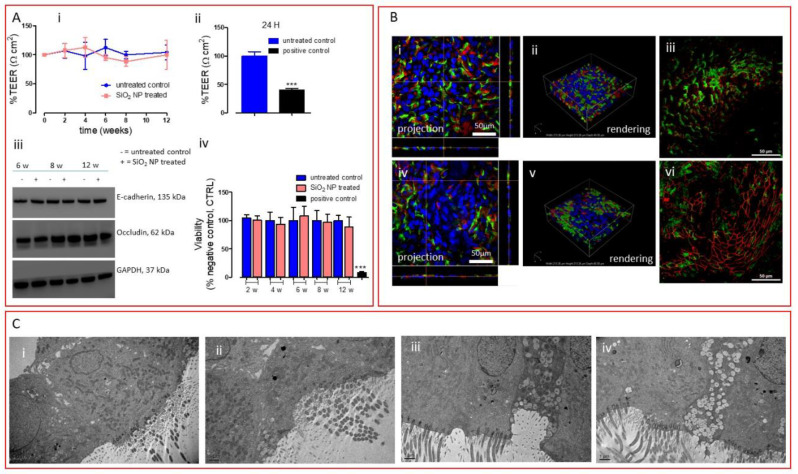
(**A**) (**i**) TEER measurements of the 3D airway model untreated or treated with repeated SiO_2_ NP aerosolization (2, 4, 6, 8, and 12 weeks). (**ii**) TEER measurement of the 3D airway model exposed to 0.3% Triton X-100 (24 h) employed as positive control. Data are expressed as mean ± standard deviation (*n* tests = 3) and show a percentage relative to the pre-exposure TEER values of each tissue. *** *p* < 0.001 vs. untreated control cells. (**iii**) Western blot analysis of membrane proteins (E-cadherin and occludin). Representative blots are shown. Quantification of Western blotting was performed with ImageJ software 1.8.0_112 (NIH, Bethesda, MD, USA). GAPDH expression was reported as protein loading control. The experiment was performed twice with comparable results. (**iv**) Viability assay (resazurin method) where 0.3% Triton X-100 was used as positive control for 24 h. Data are expressed as mean ± standard deviation (*n* tests = 3). *** *p* < 0.001 vs. untreated control cells. (**B**) Projection and rendered reconstruction of representative LSCM images of 3D bronchial epithelial cells before SiO_2_ NP exposure (**i**–**iii**) and after 12 weeks of repeated treatment (**iv**–**vi**). Cells were stained with Hoechst 33342 (nuclei, in blue, all panels), mucin 5AC antibody (goblet cells, in red, panels i, ii, iv, and v), alpha tubulin (acetyl K40) antibody (cilium cells, in green, panels **i**, **ii**, **iv**, and **v**), and ZO-1 antibody (tight junction protein, in red, panels iii and vi). Scale bars: 50 μm (63 × objective lens). (**C**) Representative TEM micrographs of the untreated tissue (**i**) and tissue repeatedly exposed to SiO_2_ NPs for (**ii**) 6 weeks, (**iii**) 8 weeks, and (**iv**) 12 weeks. Scale bars: 1 μm.

Figure 2A (i) shows that no changes in TEER were reported for the entire exposure period, suggesting that SiO_2_ NPs did not induce any barrier impairment over a long time period, compared with the positive control, which after 24 h produced a very significant TEER decrease (Figure 2A (ii)).

To further confirm the integrity of the tissue barrier, we monitored the expression of two membrane proteins, occludin, one of the integral membrane proteins specifically localized at the TJ level, and E-cadherin, a component of the adherens junctions located basally to TJ and involved in the mechanical linkage of adjacent cells [34]. Western blot analysis, reported in Figure 2A (iii), did not suggest any alteration in the expression of both proteins. Moreover, monitoring the viability of the tissue for the entire exposure period, we demonstrated that prolonged exposure to SiO_2_ NPs did not induce any cytotoxic effects to the airway tissue even after 12 weeks of exposure, as opposed to the positive control, which produced a significant decrease in viability after 24 h of exposure (Figure 2A (iv)). Continuing our in-depth analysis of the airway barrier, we move our attention to the mucociliary system. The functional components of this system are the protective mucus layer and the cilia on the surface layer, which are specialized organelles that beat to push pathogens and inhaled particles trapped in the mucus layer out of the airways [35]. Here, no visual morphological alteration of the cellular surface (on which ciliated and goblet cells are exposed) was observed after 12 weeks of exposure, as reported by LSCM images (Figure 2B (i,ii,iv,v)). Moreover, we did not observe any modification at the TJ level (Figure 2B (iii–vi)), where the expression of zonula occludens-1 (ZO-1, one of the main TJ proteins) is shown. Lastly, a structural analysis of the tissue model repeatedly exposed to SiO_2_ NPs was performed by TEM (Figure 2C). TEM images showed that 12 weeks of exposure to silica did not compromise the tissue in terms of morphology, as no significant differences were observed between the treated tissues and the untreated ones (Figure 2C (i) vs. Figure 2C (ii–iv)). However, we underline that silica particles were not found in the tissues, suggesting a rapid particle clearance by the mucociliary system, as also shown in in vivo situations [13,14,15,16]. It is worth mentioning that graphene oxide (GO) exerted a completely different impact in very similar experimental conditions but with reduced time of exposure (30 days) [22]. A decrease in TEER of about 40% was reported after 25-repeated exposure. As such reduction was associated with neither viability changes nor alteration in TJ protein expression, we ascribed TEER perturbation to subtle alterations of plasma membrane channels or pumps. This phenomenon more likely leads to an increase of GO internalization as we indeed observed. In this study, where longer exposure and higher concentrations were applied, we did not register any TEER perturbation and particle uptake, suggesting that, as opposed to GO, SiO_2_ NPs are more correctly excreted by the defense system of the airway barrier. This significantly different outcome between the two materials underlines the strength of the model, to which different nanomaterials (showing differences in physical, chemical properties) elicit different biological effects. Altogether, these data indicate that prolonged exposure to realistic doses (with relevance to occupational exposure) of SiO_2_ NPs using an advanced 3D in vitro tool does not induce any barrier damage at the condition employed. This lack of toxicity message is in line with the data provided by in vivo studies reported above, where inhalation of low doses of silica was considered [13,14,15,16]. Thus, this 3D in vitro platform (3D airway model coupled with a nebulizer system enabling the delivery of low doses of material) could be a valid alternative of in vivo models upon validation.

## 4. Conclusions

In this work, we showed an extensive characterization of the 3D airway barrier repeatedly exposed to low realistic doses of SiO_2_ NPs for 12 weeks. Indeed, the selected dose range reflects a real worker exposure lifetime to SiO_2_ NPs in a production facility. Our experimental data indicated that, at the conditions employed in this study, inhalation of SiO_2_ NPs did not produce any morphology alteration at the tissue level. The efficient mucociliary clearance system can contrast the particle uptake and its consequential negative effects on the cellular barrier integrity, as also reported by the in vivo data shown.

In conclusion, this preliminary finding suggests that this in vitro platform system (3D airway model plus aerosol system and doses simulating a real-word exposure scenario) could be a promising tool for obtaining risk-assessment-oriented data to be applied within precautionary occupational contexts. Indeed, the long-term-perspective study may pave the way for an in vitro–in vivo validation of the 3D in vitro platform and its potential applicability in animal-free testing for risk assessment of nanomaterials.
